# “Troponinosis”, the Cardiologist’s Curse—When Clinic–Laboratory Interaction Unveils the Mystery: A Case Report

**DOI:** 10.3390/jcdd10090378

**Published:** 2023-09-03

**Authors:** Davide Bosi, Simone Canovi, Andrea Pennacchioni, Pierluigi Demola, Mattia Corradini, Vincenzo Guiducci, Rossana Colla, Alessandro Navazio

**Affiliations:** 1 Cardiology Unit, AUSL—IRCCS di Reggio Emilia, 42100 Reggio Emilia, Italy; andrea.pennacchioni@ausl.re.it (A.P.); pierluigi.demola@ausl.re.it (P.D.); vincenzo.guiducci@ausl.re.it (V.G.); alessandro.navazio@ausl.re.it (A.N.); 2 Clinical Laboratory Unit, AUSL—IRCCS di Reggio Emilia, 42100 Reggio Emilia, Italy; simone.canovi@ausl.re.it (S.C.); mattia.corradini@ausl.re.it (M.C.); rossana.colla@ausl.re.it (R.C.)

**Keywords:** high-sensitivity troponin, acute coronary syndrome, immunoglobulin, laboratory tests, analytic interference

## Abstract

Cardiac troponins are key diagnostic and prognostic biomarkers in acute myocardial infarction and, more generally, for the detection of myocardial injury. Since the introduction of the first immunochemistry methods, there has been a remarkable evolution in analytical performance, especially concerning a progressive improvement in sensitivity. However, the measurement of circulating troponins remains rarely susceptible to analytical interferences. We report a case of persistently elevated troponin I concentrations in a patient with known ischemic heart disease, which almost led to unnecessary diagnostic–therapeutic interventions. A prompt laboratory consultation by the cardiologist ultimately led to the identification of an analytical interference due to troponin macrocomplexes (macrotroponin) causing elevated troponin values in the absence of a clinical presentation compatible with myocardial damage.

## 1. Introduction

Since the clinical introduction of measurement methods for cardiospecific isoforms of troponins (cTnI or T) for the detection of myocardial damage, there has been a significant improvement in their analytical performance, especially regarding improved sensitivity [1]. This has led to more accurate and earlier diagnoses of acute myocardial infarction (AMI), so much so that cTn has become a milestone in its universal definition [2]. At the same time, the increased diagnostic sensitivity has led to more frequent findings of biomarker elevations in conditions other than acute myocardial ischemia (also known as “Troponinosis”) that are acute and chronic as well as cardiac and extracardiac (e.g., sepsis, renal failure, etc.). Such elevations, often mislabeled as “false positives”, are, in fact, the expression of direct or indirect myocardial damage, by virtue of the biomarker’s excellent organ specificity [3]. However, true false-positive findings, in which no clinical and/or instrumental evidence of myocardial damage is found, are still possible and may be difficult for the clinical cardiologist to explain [4]. In this scenario, possible analytic interference must be suspected, and a laboratory consultation can be decisive.

## 2. Detailed Case Description

**Figure 1:**
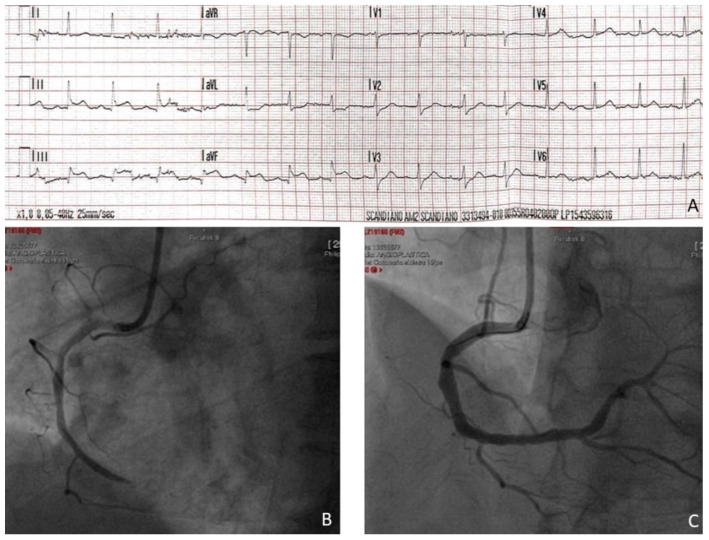
Panel (**A**): ECG diagnostic for inferior STEMI; Panel (**B**): the diagnostic coronary angiography; Panel (**C**): the final result after PTCA.

Here, we describe a case of a 60-year-old man presenting to the emergency department (ED) for sudden onset of chest pain and electrocardiographic (EKG) evidence of inferior lead ST-segment elevation (Figure 1A). On coronary angiogram (Figure 1B), a thrombotic occlusion of the distal right coronary artery (RCA) was detected and promptly treated with thromboaspiration and implantation of a sirolimus-eluting ultrathin stent (Figure 1C). The transthoracic echocardiogram showed an akinesia of the left ventricle (LV) inferior wall with a slightly reduced LV ejection fraction (LVEF) (48–50%). A single determination of circulating concentrations of cTnI (measured in plasma lithium heparin with the Siemens high-sensitivity method on a Siemens ATELLICA IM analyzer, Siemens Healthineers, Erlangen, Germany; 99th percentile for sex: 57.3 ng/L) resulted in a value of 15.994 ng/L. Plasma CK-MB was 314 ng/mL (peak value). On EKG, an evolution of the inferior leads with development of Q waves and persistence of ST-segment elevation in the subsequent 12 h was documented. The patient underwent cardiological rehabilitation with good results and was discharged without complications.

**Figure 2:**
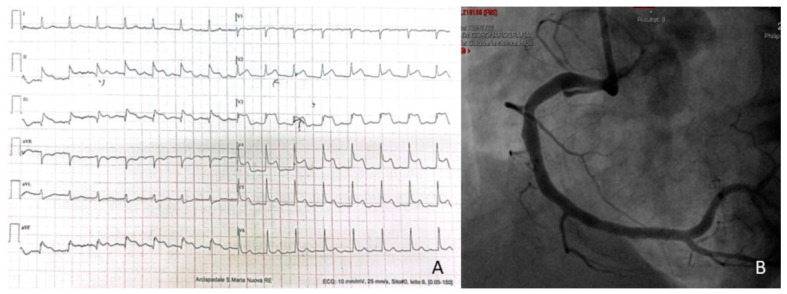
Panel (**A**): EKG showing diffuse ST-segment elevation; panel (**B**): evidence of no occlusion of the vessel and good result of previous PTCA.

After a few weeks, the patient complained of multiple episodes of stomach ache, with two new admissions to the ED. In both episodes, plasma cTnI was measured and resulted in concentrations above the 99th percentile, with a plateau trend and stable values around 700–800 ng/L. The absence of EKG and echocardiographic changes and the atypical symptoms complained of by the patient led the consulting cardiologist to withhold hospitalization, instead scheduling an outpatient follow-up visit in a short time. The follow-up visit was performed in a fully well and asymptomatic patient, confirming the stability of the scenario despite a further measurement of cTnI with values around 800 ng/L. This unusual finding did not reassure the cardiologist, who questioned the need for a definitive coronagraphic test. A few days later, the patient returned to the ED with usual epigastric pain. Echocardiogram and EKG were still unchanged from the previous tests. cTnI concentrations were still stable in serial measurements, with values around 1200 ng/L but with negative CK-MB (Table 1, summary of cTnI trend). In this case, the patient was admitted to the cardiology ward for further investigations. For persistence of abdomen discomfort on palpation in the right hypochondrium, associated with increasing cholestasis indices, a full abdomen ultra-sound examination and a subsequent computed tomography scan were performed, which raised the suspicion of an acute episode of hepatic cholangitis, later confirmed through invasive endoscopy. After suspension of Prasugrel for 5 days (3 months after acute coronary syndrome), endoscopic retrograde cholangio-pancreatography (ERCP) was successfully performed, with extraction of gallstones and the execution of a papilla sphincterotomy. A dual antiplatelet therapy (DAPT) with clopidogrel was restarted the day after. The patient’s abdominal symptoms were thus eventually attributed to the hepatobiliary disorder. Nonetheless, an explanation for the persistently elevated cTnI values was still lacking. A few days later, the patient returned to the ED once again complaining of chest pain with dynamic ST-segment elevation in the lower leads. Angiographic examination showed stent patency in the distal right coronary artery and no culprit lesions (Figure 2B). Right after the angiographic procedure, the patient reported a new episode of intense “angor” with diffuse ST-segment elevation (Figure 2A) and rapid regression. Since a vasospastic disorder was suspected, non-dihydropyridine calcium antagonist (diltiazem) therapy was immediately introduced with prompt and complete regression of symptoms. Severe anemia (hemoglobin level: 68 g/L) was subsequently diagnosed from a following test. Suspecting a complication from the previous endoscopic procedure, a follow-up esophagogastroduodenoscopy was performed and a probable post-ERCP hemorrhage was identified. Therefore, in view of the high hemorrhagic risk of the patient, a safe discontinuation of the second antiplatelet agent was chosen. Follow-up laboratory findings showed stable post-transfusion hemoglobin values, with persistently high serial concentrations of plasma cTnI (around 1200–1300 ng/L, with negative CK-MB), inconsistent with the clinical scenario. In an attempt to find an explanation, for the patient and for the cardiologists themselves, an analysis of the literature was carried out, trying to solve the issue. Could cholangitis be responsible for persistent myocardial damage? Association between biliary diseases with ECG alterations such as ST deviation, a rise in troponin values and a possible vagally mediated coronary vasospasm is described in the literature [5]. But since the latter occurred after the resolution of the cholangitis and troponin was independently altered by epigastric and chest pain, we leaned towards an alternative mechanism. Therefore, in the suspicion of analytical interference with troponin measurement, a consultancy with the laboratory staff was requested. The suspected analytical interference was investigated by the clinical laboratory with a protocol comprising method comparison, dilution testing, blocking agents and polyethylene glycol (PEG) precipitation [6]. A significant difference in cTnI concentrations measured with an alternative immunoassay confirmed the interference (187.5 ng/L, measured with Abbott Abbott Alinity i STAT high sensitive Troponin I; sex-related 99° percentile: 34.2 ng/L), even accounting for the expected bias between methods [7]. Serial dilutions of patient samples using a troponin-free diluent gave an atypical pattern of recovery, with a significant increase (taking into account the analytical imprecision) after the first dilution, but showing a linear recovery with the second one (neat: 1313 ng/L; 1/5 dilution: 1723 ng/L; 1/10 dilution: 1669 ng/L). After incubation of the sample with a heterophile blocking reagent (Heterophile Blocking Tube, Scantibodies Laboratory, Inc.), no significant variation in the measured cTnI concentrations was observed (1299 ng/L). Finally, the sample was diluted 1/2 with PEG (25% *w*/*v*), incubated for 10 min at room temperature and re-analyzed after centrifugation: a recovery of 10.6% was obtained (139 ng/L). The same PEG pretreatment resulted in a recovery of 87.8% and 91.8% in two control patient samples with concentrations of 57 ng/L and 1467 ng/L, respectively. With the alternative method, PEG pretreatment resulted in a recovery <10% (<5.1 ng/L). The analysis was then repeated seven months after both the ischemic and inflammatory problems, in a period of well-being, obtaining similar results: cTnI 723 ng/L, a significant difference with the alternative method (95.6 ng/L) and reduction in measured concentrations after precipitation with PEG (65 ng/L, with recovery of 10.1%), with definitive confirmation of the underlying analytic interference.

## 3. Discussion

**Figure 3:**
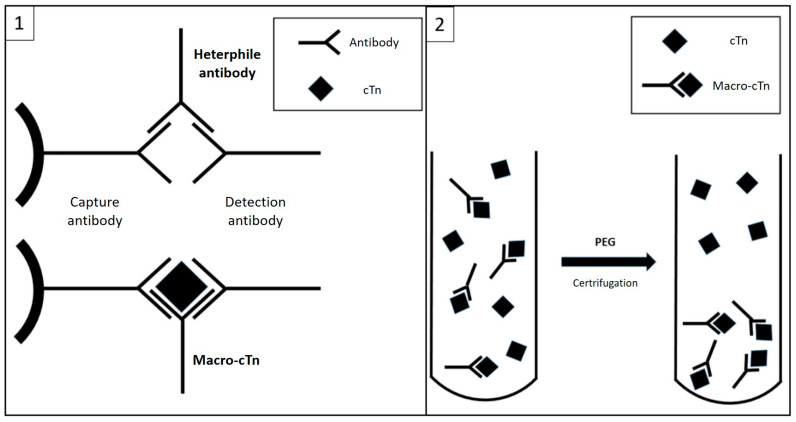
Possible biochemical mechanisms of false-positive interferences in troponin assays: 1—Heterophile antibodies may cause false-positive results by acting as a bridge between capture and detection antibodies in the absence of cTn. Macrocomplexes (macro-cTn) reduce the plasma clearance of cTn, causing an increase in the circulating concentrations of complexed molecules that are eventually measured with the immunoassay. 2—PEG treatment induces the precipitation of macrocomplexes, leaving the uncomplexed cTn molecules in the supernatant after centrifugation of the sample.

Cardiac troponins are key diagnostic and prognostic biomarkers in AMI and, more generally, for the detection of myocardial damage. However, although there has been a considerable evolution in analytical performance since the introduction of the first immunochemistry methods [8], especially regarding an improvement in sensitivity, the measurement of circulating troponins remains susceptible to analytical interference [4]. The most frequent troponin interferences described in the available literature concern the presence of heterophilic antibodies (HA) and rheumatoid factor, which, by binding to capture and/or detection antibodies, can cause falsely elevated troponin values [9,10]. Another cause is related to the formation of troponin macrocomplexes. These appear to result from binding between endogenous autoantibodies and circulating troponins, with the formation of antigen–antibody complexes that reduce plasma clearance of cTn, resulting in increased circulating concentrations [11] (Figure 3). Anti-cTn autoantibodies may be present in healthy subjects, but their presence is increased in post-AMI subjects or in patients with some form of cardiomyopathy; it is hypothesized that the release of cTn into the circulation from necrotic cardiomyocytes could trigger an auto-immune response against this molecule (in addition) [12]. In our case, it was not possible to confirm the presence of the analytical interference in cTnI concentrations before AMI, but it is equally true that it cannot be excluded that it occurred secondary to the cardiac ischemic injury. Although mainly anecdotal cases are reported in the literature [13], evidence has recently become available regarding the prevalence and clinical impact of troponin macroforms. Warner et al. report the presence of a high-molecular-weight immunoglobulin-cTnI complex in 5% of patients with elevated cTnI concentrations [14]. The study by Lam et al. [15] examining a population of 188 inpatients with elevated cTnI concentrations reports the presence of macrocomplexes in more than a half of these subjects (99/188, 52.7%); based on the numerosity of the initially selected population, a prevalence of interference from macrocomplexes was estimated by this study in 3.5% of troponin cases above the 99th percentile of the reference population [11]. Recently, Nevraumont A et al. investigated the clinical impact of interference on cardiac biomarkers. Their review, based on published cases from 1996 to 2021 and predominantly related to troponin interferences [16], reports that the finding of falsely elevated values would lead to clinical implications in 45% of established cases. These implications consist primarily of performing additional unnecessary tests (the most frequent was coronary angiography, followed by echocardiography, EKG and cardiac magnetic resonance imaging), followed by unnecessary hospitalizations and inappropriate therapeutic interventions (e.g., coronary revascularization and drug therapies such as enoxaparin and beta-blockers). In contrast, in 21% of the reviewed cases, no clinical consequences secondary to the presence of the interference were reported, while in 34% of the cases, the possible clinical consequences were not determined. The most common causes of false-positive troponin results reported in the reviewed cases were, in order, the so-called “outliers” (43%), i.e., discrepant results obtained from two repetitions of the same sample, believed to be secondary to fibrin residues or contamination of paramagnetic particles used in enzyme immunoassay methods [17], and, in line with the few case reports published in the past [10], interference due to HA (31%) and troponin macrocomplexes (10%). In the case presented here, the impact of the interference was clinically relevant. The patient had undergone several cardiology consultations and visits, risking unnecessary invasive investigations and delaying the correct diagnosis of abdominal pain, which was later revealed to be caused by cholangitis. Based on laboratory investigations, the interference was likely due to troponin macrocomplexes. The quasi-linear recovery of the analyte after serial dilutions [18] suggested the absence of interfering antibodies (e.g., HA, anti-streptavidin antibodies, etc.). HA was further excluded by use of a blocking agent. Moreover, the alternative method (Abbott) does not use biotin–streptavidin complexes, excluding both biotin and anti-streptavidin interferences. Finally, with both methods, we observed a significant reduction in cTnI after treatment with PEG, a finding that, even if not specific, is highly suggestive for the presence of macrocomplexes in this particular case. Moreover, the comparison between the two laboratory methods showed the same trend reported by other authors in the presence of macroforms, with the Siemens method more sensitive to interference than the Abbott one [19]. To date, the fluctuations around different cTnI levels (measured in the range of 700–800 ng/L or 1200–1300 ng/L) observed in various hospital cases remain unexplained. Whether this is due to variations in the kinetics of immune complex formation is currently unknown, due to the paucity of available evidence.

## 4. Conclusions

This case highlights the relevance of comprehensive patient assessment, emphasizing the importance of serial troponin sampling and close collaboration with the clinical laboratory. Cardiac biomarker results must always be contextualized and interpreted as part of the clinical puzzle: in the case of persistently elevated but stable values (i.e., without the typical rise and fall kinetics) and in the absence of other data suggestive of myocardial damage, the clinician must raise the suggestion of analytic interference. Being aware of it, and remembering that interferences other than HA could be present, can facilitate communication with the laboratory and avoid both dangerous consequences for the patients and additional unnecessary costs.

## Figures and Tables

**Table 1:** Timeline of troponin trend.

	Clinical Event	Laboratory Test
**Week 0**	Inferior STEMI	Hs-TnI 15,993 ng/L (peak) CK-MB 314 ng/mL (peak)
**Week 4**	Chest pain-emergency department	Hs-TnI 727 ng/L
**Week 6**	Chest pain-emergency department	Hs-TnI 854 – 868 ng/L CK-MB 1.37 ng/mL
**Week 7**	Physical examination-asymptomatic	Hs-TnI 886 ng/L
**Week 10**	Cholangitis	Hs-TnI 1261 ng/L CK-MB 1.66 ng/mL
**Week 12**	AMI type 2-suspected vasospatic angina	Hs-TnI 1316 ng/L CK-MB 3.11 ng/mL
**Week 28**	Physical examination-asymptomatic	Hs-TnI 723 ng/L

## Data Availability

Data are available upon special request.
